# Living with a transplanted liver is associated with cytopenias: a nationwide cohort study

**DOI:** 10.3389/fgstr.2025.1543618

**Published:** 2025-08-06

**Authors:** Kajinth Manogarathaas, Nicoline S. Arentoft, Jens G. Hillingsø, Anne Marie R. Jensen, Annette D. Fialla, Gerda E. Villadsen, Peter Holland-Fischer, Shoaib Afzal, Børge G. Nordestgaard, Peter Brown, Allan Rasmussen, Susanne D. Nielsen

**Affiliations:** ^1^ Department of Infectious Diseases, Copenhagen University Hospital - Rigshospitalet, Copenhagen, Denmark; ^2^ Department of Surgery and Transplantation, Copenhagen University Hospital – Rigshospitalet, Copenhagen, Denmark; ^3^ Department of Gastroenterology, Odense University Hospital, Odense, Denmark; ^4^ Department of Hepatology and Gastroenterology, Aarhus University Hospital, Aarhus, Denmark; ^5^ Department of Gastroenterology, Aalborg University Hospital, Aalborg, Denmark; ^6^ The Copenhagen General Population Study, Department of Clinical Biochemistry, Copenhagen University Hospital - Herlev and Gentofte, Herlev, Denmark; ^7^ Department of Clinical Medicine, Faculty of Health and Medical Sciences, University of Copenhagen, Copenhagen, Denmark; ^8^ Department of Hematology, Copenhagen University Hospital - Rigshospitalet, Copenhagen, Denmark

**Keywords:** cytopenia, anemia, neutropenia, lymphocytopenia, thrombocytopenia, liver transplantation, LTX, liver transplant receipients

## Abstract

Hematological abnormalities are common in liver transplant recipients, but evidence beyond the first-year post-transplantation is scarce. We aimed to evaluate hematological abnormalities in liver transplant recipients beyond the first-year post-transplantation. We included 437 liver transplant recipients and 1,744 age- and sex-matched controls from the general population. Odds for cytopenias were assessed using logistic regression analyses adjusted for age, sex, ethnicity, hs-CRP, smoking, and alcohol use. Potential transplant-related risk factors were assessed in liver transplant recipients only. The median time since transplantation was 7.8 years, and 47% had autoimmune liver disease as the indication for transplantation. Compared to controls, liver transplant recipients had a higher prevalence of anemia (24.5% vs. 3.5%), neutropenia (2.1% vs. 0.1%), lymphocytopenia (18.4% vs. 1.5%), and thrombocytopenia (19.2% vs. 2.2%). Living with a transplanted liver was independently associated with higher odds of anemia (aOR, 7.84 [95% CI: 5.04 – 12.18], *p*<0.001), lymphocytopenia (aOR 16.69 [95% CI: 9.56 – 29.12], *p*<0.001), and thrombocytopenia (aOR 10.19 [95% CI: 6.07 – 17.13], *p*<0.001). No association was found between cytopenias, specific types of immunosuppressive treatments, or cirrhosis at transplantation. In conclusion, hematological abnormalities are common in liver transplant recipients, even several years post-transplantation, and increased attention towards cytopenia in this population is warranted.

## Introduction

Hematological abnormalities are common among liver transplant recipients within the first-year post-transplantation (1–4), with anemia and thrombocytopenia being the most frequently reported cytopenias (3, 5–12). While cytopenias are highly prevalent in the early period post-transplantation, evidence beyond the first year is scarce. Post-transplant cytopenia can arise from various risk factors, including commonly used immunosuppressive medications like mycophenolate mofetil, tacrolimus, and prednisolone (13–18), as well as valganciclovir for CMV prophylaxis and sulfamethoxazole-trimethoprim for PCP prophylaxis (19–22). Additionally, factors such as increasing age, male sex, hypersplenism, impaired renal function affecting erythropoietin production, and infections may also contribute to cytopenia (6, 7, 23, 24).

One previous study found higher hemoglobin and thrombocyte counts 18 months post-transplantation compared to pre-transplantation levels in cirrhotic patients (25), but no studies have compared the prevalence of cytopenia in liver transplant recipients to that in the background population.

The objectives of this study were to determine the prevalence of anemia, neutropenia, lymphopenia, and thrombocytopenia in liver transplant recipients beyond the first-year post-transplantation compared to controls from the general population. Furthermore, we aimed to identify potential risk factors associated with cytopenias in liver transplant recipients. We hypothesized that the prevalence of cytopenia is higher in liver transplant recipients beyond the first-year post-transplantation than in the general population and that increasing age and treatment with immunosuppressive medications are risk factors for cytopenias, while increasing time since transplantation is associated with lower risk of cytopenias in liver transplant recipients.

## Materials and methods

### Study design and participants

The Danish Comorbidity in Liver Transplant Recipients (DACOLT) study is an ongoing prospective cohort study of liver transplant recipients that was initiated in 2021. The overall aim of the DACOLT study is to assess the burden of comorbidities in liver transplant recipients and the associated risk factors (26). All liver transplant recipients >20 years with residency in Denmark, who were able to give informed consent, were invited to participate in the study. The current study included all liver transplant recipients included in the DACOLT study before December 1^st^, 2023, who had at least one available measurement of hematology and were ≥1-year post-transplantation. Participants were followed at outpatient clinics at one of four regional hospitals in Denmark: Aalborg University Hospital, Aarhus University Hospital, Odense University Hospital, and Copenhagen University Hospital – Rigshospitalet. Controls were recruited from the Copenhagen General Population Study (CGPS), a prospective cohort study with >110.000 participants aged 20-100 years included from the general population in the greater Copenhagen area (27, 28).

Participants in both the DACOLT study and the CGPS completed a questionnaire regarding health and medication. Data from questionnaires included information on ethnicity, use of alcohol, and smoking habits, including current smoking status and cumulated smoking history, and educational level. Ethnicity was self-reported and defined according to grandparents’ country of origin. The response options were as follows: ‘Danish’, ‘Other Scandinavian’, ‘Other European’ or ‘Other Ethnicities’. If any grandparent had a different ethnicity, ‘Other Ethnicities’ was selected. Use of alcohol was categorized as one of the following: Never, monthly, weekly, or daily consumption of alcohol. Smoking status was defined as one of the following: Never, current, former, or unknown. Pack years was defined as number of years smoking 20 cigarettes per day.

Both the DACOLT study (H-20052199) and the CGPS (H-KF-01-144/01) received approval from the Ethics Committee of the Capital Region, Denmark. The DACOLT study has been registered at ClinicalTrials.gov (NCT04777032). All participants provided oral and written informed consent. The studies were conducted according to the Declaration of Helsinki.

### Liver transplantation variables

Information regarding the use of immunosuppressive medication at time of inclusion in the DACOLT study was retrieved from medical records and the Shared Medication Record (FMK), a national health registry that contains all medical prescriptions authorized by healthcare professionals for citizens in Denmark (29). All liver transplant-related data, including the indication for transplantation and date of transplantation were acquired from review of the participants’ electronic medical records. Cirrhosis at the time of transplantation was defined according to the histological examination of the explant. Lack of energy was defined as “Yes” to the question “Have you felt lacking in energy or strength?”. Breathlessness was defined as “Yes” to the question “Do you get very breathless when walking uphill or climbing one flight of stairs?

### Blood sample collection

Blood samples from liver transplant recipients in the DACOLT study and CGPS participants including measurements of hemoglobin, mean corpuscular volume (MCV), mean corpuscular hemoglobin concentration (MCHC), absolute neutrophil count (ANC), lymphocytes, thrombocyte count, and high-sensitivity C-reactive protein (hs-CRP) were analyzed as routine biochemistry.

### Outcome definitions

All cytopenias were defined according to the Common Terminology Criteria for Adverse Events (CTCAE) version 5.0 (30). Anemia was defined as low hemoglobin concentration (male: < 8.3 mmol/L; female: < 7.3 mmol/L) and classified as mild (male: 6.2 – 8.3 mmol/L; female: 6.2 – 7.3 mmol/L), moderate (4.9 – 6.1 mmol/L), and severe (< 4.9 mmol/L). MCV was classified as: microcytic (≤ 84 fL), normocytic (85 – 100 fL), and macrocytic (> 100 fL). MCHC was classified as: hypochromatic (≤ 19.6 mmol/L), normochromatic (19.7 – 22.2 mmol/L), and hyperchromatic (> 22.2 mmol/L). Neutropenia was defined as low neutrophil granulocytes < 1.5 x 10^9^ cells/L and classified as mild (1.0 – 1.5 x 10^9^ cells/L), moderate (0.5 – 0.9 x 10^9^ cells/L), and severe (< 0.5 x 10^9^ cells/L). Lymphocytopenia was defined as low lymphocytes < 1.0 x 10^9^ cells/L) and classified as: mild (0.8 – 1.0 x 10^9^ cells/L), moderate (0.5 – 0.79 x 10^9^ cells/L), and severe (<0.5 x 10^9^ cells/L). Thrombocytopenia was defined as low thrombocytes < 150 x 10^9^ cells/L and classified as mild (100 – 150 x 10^9^ cells/L), moderate (75 – 99 x 10^9^ cells/L), and severe (< 75 x 10^9^ cells/L).

### Statistics

Liver transplant recipients were matched on sex and 1-year age intervals with controls, aiming for a matching ratio of 1:4. However, for two liver transplant recipients it was only possible to match with two controls. Descriptive statistics were used to assess differences between liver transplant recipients and controls. For continuous data, t-tests or the Wilcoxon rank sum test were performed for normally or non-normally distributed variables, respectively. Pearson’s chi-squared test was used for categorical variables.

The prevalence of cytopenias was compared between liver transplant recipients and controls using Pearson’s chi-squared test. The association between living with a transplanted liver and cytopenia was explored using logistic regression for each of the four dependent categorical variables: anemia, neutropenia, lymphocytopenia, and thrombocytopenia. The association between living with a transplanted liver and hemoglobin, neutrophile granulocytes, lymphocytes, and thrombocytes were assessed using linear regression analyses. In both the linear and logistic regression analyses, living with a transplanted liver was included as the independent variable in a base model adjusted for age and sex, and a fully adjusted model adjusted for age, sex, ethnicity, hs-CRP, smoking, and use of alcohol. To test the robustness of our findings of the association between living with a transplanted liver and cytopenias, we performed two stratified analyses: only comparing liver transplant recipients with cirrhosis at time of transplantation with controls and excluding liver transplant recipients with autoimmune liver disease as reason for transplantation. In exploratory analyses, we used logistic regression to investigate if anemia in liver transplant recipients was associated with more lack of energy and more breathlessness. In the combined cohort, potential risk factors for cytopenias were assessed using logistic regression both in univariate models and a model including all potential risk factors. The following risk factors were investigated: Liver transplantation, age, sex, ethnicity, hs-CRP, smoking, and alcohol.

Among liver transplant recipients, potential transplant-related risk factors for cytopenias were assessed using multiple logistic regression adjusted for age and sex with potential risk factors included in the model one at a time. The following possible risk factors were investigated: use of immunosuppressive medication (mycophenolate mofetil/azathioprine/none; tacrolimus/everolimus/cyclosporine/none; prednisolone, yes/no), cirrhosis at time of transplantation (yes/no), time since transplantation and indication for transplant (autoimmune liver disease, yes/no). To assess differences in the prevalence of anemia, neutropenia, lymphocytopenia and thrombocytopenia between liver transplant recipients with and without autoimmune liver disease, Pearson’s chi-square test was used. To investigate whether trough levels of tacrolimus were associated with the presence of cytopenias, we conducted logistic regression analyses with a base model and fully adjusted model.

A two-sided *p*-value < 0.05 was considered significant. The statistical analysis was conducted in R studio 2023.09.0 build 463 with R 4.3.2 for Windows 10 Enterprise.

## Results

This study included 437 liver transplant recipients and 1744 controls. The median age for liver transplant recipients and controls was 55.4 and 55.3 years, respectively (**Table 1**). Both groups were predominantly males of Scandinavian descendent, but liver transplant recipients displayed a greater ethnic diversity than controls. Liver transplant recipients also had lower educational level than controls and were more likely to abstain from alcohol (**Table 1**).

**Table 1:** Characteristics of liver transplant recipients and controls.

Characteristics	Liver transplant recipients (n = 437)	Controls (n = 1744)	*p*-value
Sex (male), n (%)	234 (53.5)	862 (49.4)	0.14
Age, years, median (IQR)	55.4 [45.2 - 64.6]	55.3 [45.5 - 63.7]	0.96
Ethnicity, n (%) Danish Other Scandinavian Other European Other Ethnicities	342 (79.2) 16 (3.7) 43 (10.0) 29 (7.2)	1510 (87.6) 81 (4.7) 112 (6.5) 22 (1.3)	<0.001
Education level No education Short education (up to 3 yrs with books) Vocational or equivalent (1- 3 yrs) Medium-length education (3 yrs) University education	50 (11.8) 41 (9.6) 161 (37.9) 98 (23.1) 75 (17.6)	122 (7.0) 161 (9.3) 461 (26.6) 528 (30.4) 462 (26.6)	<0.001
Alcohol, n (%) Never Monthly Weekly Daily	274 (65.4) 84 (20.0) 46 (11.0) 15 (3.6)	249 (14.4) 548 (31.6) 637 (36.7) 301 (17.3)	<0.001
Smoking status, n (%) Never Current Former	249 (57.0) 51 (11.7) 137 (31.4)	902 (51.7) 218 (12.5) 624 (35.8)	0.14
Cumulated smoking history, pack years, median (IQR)	11.2 [4.8 - 20.0]	12.0 [5.0 - 25.0]	0.29
Hemoglobin, mmol/L, median (IQR)	8.5 [7.9 - 9.2]	8.8 [8.3 - 9.3]	<0.001
Neutrophil granulocytes, x 10^9^ cells/L, median (IQR)	3.6 [2.7 - 4.8]	3.8 [3.1 - 4.7]	0.009
Lymphocytes, x 10^9^ cells/L, median (IQR)	1.6 [1.1 - 2.0]	2.0 [1.6 - 2.4]	<0.001
Thrombocytes, x 10^9^ cells/L, median (IQR)	197 [162 - 247]	243 [207 - 280]	<0.001
Time since transplantation (years), median (IQR)	7.8 [4.2 - 14.5]	–	–
Cirrhosis at time of transplantation (yes), n (%)	276 (63.2)	–	–
Reason for transplantation, n (%) Autoimmune liver diseases Autoimmune hepatitis Primary biliary cholangitis Primary sclerosing cholangitis Cirrhosis (alcohol and cryptogenic) Alcoholic cirrhosis Cryptogenic cirrhosis Hepatocellular carcinoma Fulminant hepatic failure Metabolic liver diseases Hepatitis C Other Polycystic liver disease Hepatitis B Amyloidosis Biliary atresia MASLD or MASH Other liver diseases	207 (47.4) 50 (11.4) 43 (9.8) 132 (30.2) 75 (17.2) 50 (11.4) 25 (5.7) 28 (6.4) 35 (8.0) 22 (5.0) 14 (3.2) 94 (21.5) 22 (5.0) 6 (1.4) 9 (2.1) 14 (3.2) 5 (1.1) 40 (9.2)	–	–
Immunosuppressive medication at time of inclusion, n (%) Tacrolimus Cyclosporine Everolimus Azathioprine Mycophenolate mofetil (MMF) Prednisolone	354 (81.0) 48 (11.0) 36 (8.2) 52 (11.9) 302 (69.1) 184 (42.1)	–	–

Median time since transplantation was 7.8 years [interquartile range (IQR), 4.2 – 14.5] (**Table 1**). At time of transplantation, 275 participants (62.9%) had cirrhosis, regardless of the underlying cause. Autoimmune liver disease was the most frequent indication for liver transplantation in 207 (47.4%) liver transplant recipients. Most liver transplant recipients were treated with tacrolimus and mycophenolate mofetil (81.0 and 69.1%, respectively), and 184 (42.1%) also received prednisolone at the time of inclusion in the study.

### Prevalence of cytopenia in liver transplant recipients and controls

**Figure 1:**
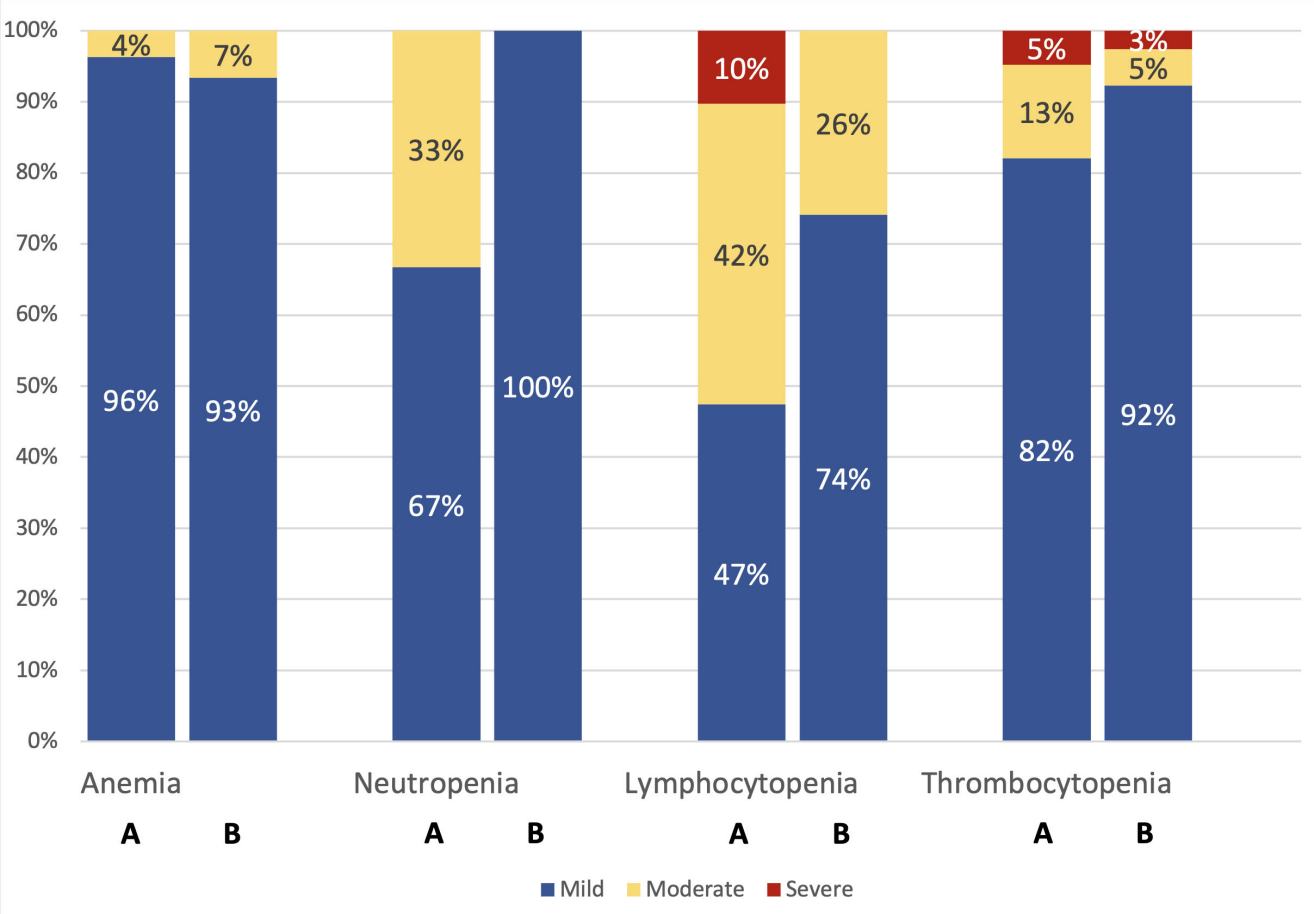
For each cytopenia (anemia, neutropenia, lymphocytopenia, and thrombocytopenia), bar A represents liver transplant recipients, and bar B represents controls. The severity of cytopenia, expressed as a percentage, was assessed only among participants with cytopenia. P-values for comparison between liver transplant recipients and controls: Anemia: p = 0.65; Neutropenia: p = 1.00; Lymphocytopenia: p = 0.03; Thrombocytopenia: p = 0.33.

Liver transplant recipients had a lower hemoglobin concentration than controls (median IQR, 8.5 mmol/L [7.9 – 9.2] vs 8.8 mmol/L [8.3 – 9.3], *p*<0.001), and a higher prevalence of anemia. (24.5% vs 3.5%, *p*<0.001). However, no significant differences were found in the severity of anemia between liver transplant recipients and controls with anemia (**Figure 1**). Among participants with anemia, the prevalence of hypochromatic anemia was higher in liver transplant recipients than in controls (25.0% vs 8.2%, *p=*0.014), while the distribution of MCV was not significantly different between liver transplant recipients and controls. In logistic regression analysis adjusted for age and sex, anemia was associated with a borderline significant increase in odds of reporting lack of energy (OR 1.8 [95% CI: 0.97–3.33], *p* = 0.06). However, no significant association was found between anemia and breathlessness (OR 1.03 [95% CI: 0.60–1.77], *p* = 0.91).

The median neutrophil count was lower in liver transplant recipients (median IQR, 3.6 x 10^9^ cells/L [2.7 – 4.8] vs 3.8 x 10^9^ cells/L [3.1 – 4.7], *p*=0.009), with a higher prevalence of neutropenia (2.1% vs 0.1%, *p*<0.001). However, no significant differences were found in the severity of neutropenia between liver transplant recipients and controls with neutropenia (**Figure 1**).

The median lymphocytes count was lower in liver transplant recipients (median IQR, 1.6 x 10^9^ cells/L [1.1 – 2.0] vs 2.0 [1.6 – 2.4], *p*<0.001), with a higher prevalence of lymphocytopenia. (18.4% vs 1.5%, *p*<0.001). Among those with lymphocytopenia, liver transplant recipients had more severe cases than controls (**Figure 1**).

The median thrombocyte count was lower in liver transplant recipients (median IQR, 197 x 10^9^ cells/L [162 – 247] vs 243 x 10^9^ cells/L [207 – 280], *p*<0.001), with a higher prevalence of thrombocytopenia (19.2% vs 2.2%, *p*<0.001). However, no significant differences were found in the severity of thrombocytopenia (**Figure 1**).

**Figure 2:**
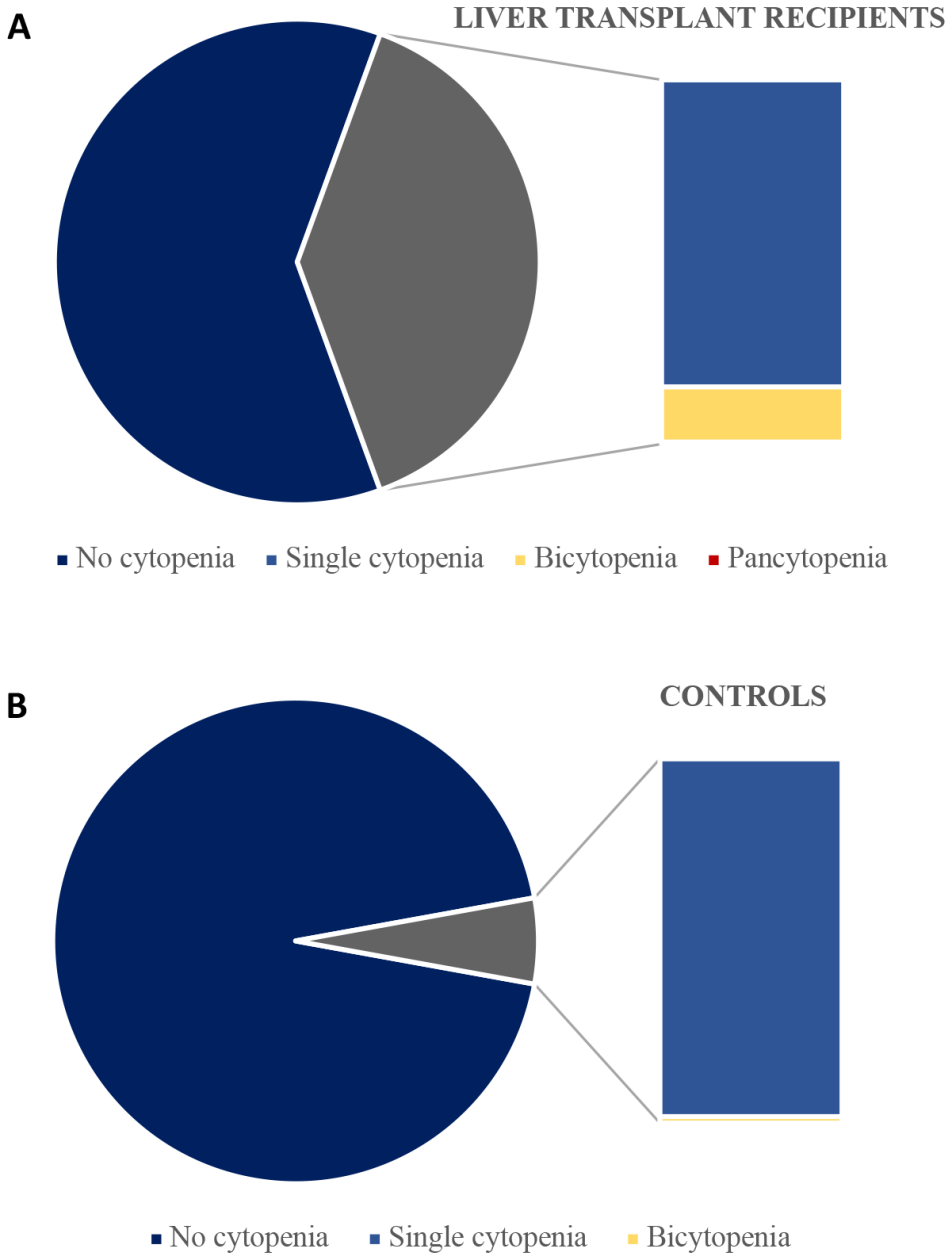
Prevalence of single-, bi- and pancytopenia among liver transplant recipients **(A)** and controls with complete data **(B)**.

The prevalence of both single cytopenia and bicytopenia were higher in liver transplant recipients than in controls (**Figure 2**). While majority of both groups had no cytopenia, 32.8% of liver transplant recipients had a single cytopenia compared to 5.6% in controls (**Table 2**). Bicytopenia was present in 25 (5.9%) liver transplant recipients, while this was only the case for one of the controls (0.1%). Pancytopenia was only found in one liver transplant recipient.

**Table 2:** Prevalence of single-, bi- and pancytopenia among liver transplant recipients and controls with complete data.

Cytopenia status	Liver transplant recipients (n = 424)	Controls (n = 1740)	p-value
Cytopenia			<0.001
No cytopenia, No. (%)	259 (61.1)	1.642 (94.4)	
Single cytopenia, No. (%)	139 (32.8)	97 (5.6)	
Bicytopenia, No. (%)	25 (5.9)	1 (0.1)	
Pancytopenia, No. (%)	1 (0.2)	0 (0.0)	

### Risk factors associated with cytopenia in liver transplant recipients and controls

Living with a transplanted liver was associated with higher odds of anemia, lymphocytopenia, and thrombocytopenia both in the base model and after adjustment for age, sex, ethnicity, hs-CRP, smoking, and alcohol (adjusted odds ratio (aOR), 7.84 [95% confidence interval (CI): 5.04 – 12.18], *p*<0.001 for anemia, aOR 16.69 [95% CI: 9.56 – 29.12], *p*<0.001 for lymphocytopenia), and aOR 10.19 [95% CI: 6.07 – 17.13], *p*<0.001 for thrombocytopenia, **Table 3**). Living with a transplanted liver was associated with neutropenia in the base model adjusted for age and sex (OR 37.71 [4.76 – 298.50], *p*<0.001, **Table 3**). Adjusted analyses for neutropenia were not feasible as only one of the controls had neutropenia. These findings were further supported by our linear regression analyses among liver transplant recipients (**Table 4**).

**Table 3:** Odds ratios for cytopenia comparing liver transplant recipients with controls.

Cytopenia type	OR for cytopenia comparing liver transplant recipients with controls			
	Model 1 OR (95% CI)	*p-*value	Model 2 OR (95% CI)	*p-*value
Anemia	9.17 [6.52 – 12.89]	<0.001	7.84 [5.04 – 12.18]	<0.001
Neutropenia	37.71 [4.76 – 298.50] *	<0.001	–	–
Lymphocytopenia	14.56 [9.24 – 22.95]	<0.001	16.69 [9.56 – 29.12]	<0.001
Thrombocytopenia	10.30 [6.92 – 15.33]	<0.001	10.19 [6.07 – 17.13]	<0.001

Model 1: Base model adjusted for age and sex.

Model 2: Fully adjusted model for age, sex, ethnicity, hs-CRP, smoking, and alcohol.

*Adjusted analyses were not possible for neutropenia, as only one controls had neutropenia. Results for neutropenia are presented for unadjusted analyses.

**Table 4:** Linear regression analysis of hemoglobin, neutrophile granulocytes, lymphocytes, and thrombocytes in liver transplant recipients.

Variable	Model 1 OR (95% CI)	*p-*value	Model 2 OR (95% CI)	*p-*value
Hemoglobin (Intercept)	8.63 [8.52; 8.75]	<0.001	8.59 [8.43; 8.75]	<0.001
Liver Transplantation (yes/no)	-0.31 [-0.38; -0.24]	<0.001	-0.28 [-0.37; -0.20]	<0.001
Neutrophil granulocyte (Intercept)	3.90 [3.65; 4.15]	<0.001	3.70 [3.37; 4.03]	<0.001
Liver Transplantation (yes/no)	-0.05 [-0.20; 0.10]	0.52	-0.33 [-0.51; -0.15]	<0.001
Lymphocytes (Intercept)	2.43 [2.31; 2.56]	<0.001	2.33 [2.16; 2.51]	<0.001
Liver Transplantation (yes/no)	-0.39 [-0.47; -0.32]	<0.001	-0.45 [-0.54; -0.35]	<0.001
Thrombocytes (Intercept)	256.98 [246.73; 267.23]	<0.001	261.61 [247.45; 275.77]	<0.001
Liver Transplantation (yes/no)	-36.85 [-43.04; -30.65]	<0.001	-38.59 [-46.14; -31.04]	<0.001

Model 1: Base model adjusted for age and sex.

Model 2: Fully adjusted model for age, sex, ethnicity, hs-CRP, smoking, and alcohol.

Increasing age was associated with higher odds for anemia and lymphocytopenia in both univariate models and the fully adjusted model with potential risk factors (**Supplementary Table 1**). Male sex was associated with higher odds for anemia and thrombocytopenia in univariate analysis, however only remained significant for anemia in the fully adjusted model. Higher hs-CRP was associated with higher odds for lymphocytopenia in both univariate analyses and the fully adjusted model (**Supplementary Table 1**). No other factors were found to be significantly associated with cytopenias.

### Liver transplantation related risk factors associated with cytopenias

No significant associations were found between specific types of immunosuppressive medication and cytopenia in analyses only including liver transplant recipients (**Table 5**). A shorter time since transplantation was associated with higher odds of anemia (OR 2.49 [95% CI: 1.56 – 3.99], *p*<0.001) and neutropenia (OR 4.48 [95% CI: 1.09 – 18.36], *p*<0.04) for participants between 1 – 5 years post-transplantation compared to >5 years post-transplantation in the base model after adjustment for age and sex and remained significant for anemia after full adjustment. No significant associations were found between cytopenias and the reason for transplantation or presence of cirrhosis at the time of transplantation. As a sensitivity analyses, we assessed the prevalence of cytopenias among liver transplant recipients with cirrhosis at time of transplantation compared to controls. Cytopenias were significantly more prevalent across all categories in liver transplant recipients. Anemia was observed in 26.5% of liver transplant recipients with cirrhosis at the time of transplantation compared to 3.5% of controls (*p* < 0.001), neutropenia in 2.2% versus 0.1% (*p* < 0.001), lymphocytopenia in 19.7% versus 1.5% (*p* < 0.001), and thrombocytopenia in 20.7% versus 2.2% (*p* < 0.001). The association between living with a transplanted liver and cytopenias remained consistent in analyses limited to patients with cirrhosis and in those excluding autoimmune liver disease as the indication for transplantation (**Supplementary Tables 3**, **4**).

**Table 5:** Transplant-related risk factors for cytopenia in liver transplant recipients.

Characteristics	Anemia						Neutropenia			
	Model 1	*p*-value		Model 2		*p*-value	Model 1	*p*-value	Model 2	*p*-value
Immunosuppressive medication										
* MMF*	*Reference*			*Reference*			*-*		-	
* AZA*	0.52 [0.22 - 1.22]	0.14		0.54 [0.21 – 1.40]		0.20				
* None*	1.11 [0.63 - 1.97]	0.72		1.14 [0.60 – 2.16]		0.68				
Immunosuppressive medication										
* Tacrolimus*	*Reference*			*Reference*			*-*		*-*	
* Cyclosporine*	0.99 [0.49 - 2.01]	0.98		0.82 [0.35 – 1.89]		0.63				
* Everolimus*	2.21 [1.00 - 4.88]	0.05		1.82 [0.73 – 4.53]		0.20				
* None*	0.63 [0.07 - 5.58]	0.68		0.78 [0.08 – 7.34]		0.83				
Prednisolone, yes vs no	1.26 [0.80 - 1.97]	0.32		1.50 [0.89 – 2.52]		0.13	1.68 [0.44 - 6.38]	0.45		
Cirrhosis, yes vs no	1.26 [0.71 - 2.22]	0.43		1.24 [0.62 – 2.46]		0.54	0.86 [0.10 - 7.50]	0.89		
Time since transplantation										
* > 5 years*	*Reference*			*Reference*			*Reference*		*-*	
* 1-5 years*	2.49 [1.56 - 3.99]	<0.001		2.77 [1.60 – 4.79]		<0.001	4.48 [1.09 - 18.36]	0.04		
Reason for transplantation										
* Autoimmune*	*Reference*			*Reference*			*Reference*		*-*	
* Other*	0.81 [0.52 - 1.26]	0.35		0.82 [0.48 – 1.39]		0.46	3.64 [0.74 - 17.88]	0.11		
Characteristics	Lymphocytopenia						Thrombocytopenia			
	Model 1		*p*-value	Model 2	*p*-value		Model 1	*p*-value	Model 2	*p*-value
Immunosuppressive medication										
* MMF*	*Reference*			*Reference*			*Reference*		*Reference*	
* AZA*	1.24 [0.59 - 2.61]		0.58	1.18 [0.50 – 2.80]	0.71		1.00 [0.46 - 2.14]	0.99	1.21 [0.51 – 2.85]	0.67
* None*	0.68 [0.33 - 1.37]		0.28	0.73 [0.34 – 1.59]	0.43		1.14 [0.61 - 2.11]	0.68	1.01 [0.50 – 2.04]	0.99
Immunosuppressive medication										
* Tacrolimus*	*Reference*			*Reference*			*Reference*		*Reference*	
* Cyclosporine*	1.52 [0.73 - 3.17]		0.26	1.68 [0.73 – 3.89]	0.22		1.15 [0.53 - 2.47]	0.72	1.09 [0.45 – 2.68]	0.85
* Everolimus*	1.02 [0.37 - 2.80]		0.97	0.95 [0.29 – 3.10]	0.93		0.47 [0.14 - 1.60]	0.23	0.27 [0.06 – 1.24]	0.09
* None*	2.23 [0.38 - 13.12]		0.38	3.89 [0.54 – 28.31]	0.18		2.14 [0.37 - 12.41]	0.40	1.96 [0.31 – 12.32]	0.47
Prednisolone, yes vs no	1.67 [1.02 - 2.76]		0.04	1.65 [0.93 – 2.92]	0.09		0.98 [0.60 - 1.59]	0.93	1.09 [0.62 – 1.92]	0.76
Cirrhosis, yes vs no	0.61 [0.29 - 1.28]		0.19	0.78 [0.34 – 1.80]	0.55		1.02 [0.53 - 1.97]	0.96	1.03 [0.47 – 2.27]	0.94
Time since transplantation										
* > 5 years*	*Reference*			*Reference*			*Reference*		*Reference*	
* 1-5 years*	1.43 [0.84 - 2.41]		0.19	1.31 [0.71 – 2.42]	0.39		1.09 [0.65 - 1.81]	0.75	1.16 [0.65 – 2.07]	0.63
Reason for transplantation										
* Autoimmune*	*Reference*			*Reference*			*Reference*		*Reference*	
* Other*	1.11 [0.67 - 1.83]		0.68	1.62 [0.90 – 2.94]	0.11		1.21 [0.75 - 1.97]	0.44	1.24 [0.70 – 2.20]	0.45

Mycophenolate mofetil, MMF; Azathioprine, AZA.

Model 1: Base model adjusted for age and sex. Model 2: Fully adjusted model for age, sex, ethnicity, hs-CRP, smoking, and alcohol.

We found no statistically significant associations between tacrolimus trough levels and anemia, thrombocytopenia, and any type of cytopenia (**Supplementary Table 5**). In contrast, a borderline significant association with higher OR for lymphocytopenia was seen when tacrolimus trough levels increased (aOR 6.94 [95% CI: 0.71–67.62], *p* = 0.10). Furthermore, no significant differences were found in the prevalence of anemia, neutropenia, lymphocytopenia, and thrombocytopenia between liver transplant recipients with and without autoimmune liver disease (**Table 6**).

**Table 6:** Prevalence of anemia, neutropenia, lymphocytopenia and thrombocytopenia in patients with and without autoimmune liver disease.

Cytopenia type	Autoimmune liver disease (n = 207)	Other cause (n = 230)	Total (n = 437)	p-value
Prevalence of anemia	53 (25.6%)	54 (23.5%)	107 (24.5%)	0.69
Prevalence of neutropenia	201 (99.0%)	214 (96.8%)	415 (97.9%)	0.22
Prevalence of lymphocytopenia	168 (82.4%)	179 (81.0%)	347 (81.6%)	0.81
Prevalence of thrombocytopenia	171 (82.6%)	182 (79.1%)	353 (80.8%)	0.42

Our analyses found no significant differences between the groups.

## Discussion

We found that living with a transplanted liver was independently associated with higher odds of anemia, lymphocytopenia and thrombocytopenia compared to controls from the general population. Male sex and shorter time since transplantation was associated with higher odds for anemia, while higher age was associated with anemia and lymphocytopenia, and higher levels of hs-CRP were associated with lymphocytopenia. No association was found between cytopenias and specific types of immunosuppressive treatments or cirrhosis at the time of transplantation.

The prevalence of anemia was higher in liver transplant recipients than controls and living with a transplanted liver was an independent risk factor for anemia. Furthermore, we found that male sex and shorter time since transplantation were associated with an increased risk of anemia. These results were consistent with other studies, in which even higher prevalence of anemia was observed one- and two-years post-transplantation, respectively (3, 23). However, the lack of consensus definition for anemia poses a challenge in comparing our results to existing literature. The severity of anemia was most commonly mild in both liver transplant recipients and controls with anemia with a similar distribution of anemia severity in the two groups. However, liver transplant recipients had more cases of hypochromatic anemia compared to controls. This finding is consistent with data from a prior study (3), which additionally found no significant impact of late onset of anemia on mortality or graft failure three years post-transplantation. The larger proportion of hypochromatic anemia may indicate iron deficiency that may, in part, be explained by underlying factors such as hemolysis, inflammation, malabsorption, or impaired utilization of iron post-transplantation (23, 31). Although, other causes of anemia such as bone marrow dysfunction cannot be ruled out. However, existing evidence has already identified hemoglobin levels as an independent risk factor for cardiovascular events, ischemic stroke and long-term mortality in patients with acute myocardial infarction (32–35). Overall identifying risk factors such as male sex and shorter time since transplantation to be associated with an increased risk of anemia may aid physicians on who to prioritize to proactively monitor at-risk patients with early intervention if necessary. While anemia in the general population is associated with impaired cognitive function, fatigue, cardiovascular strain, and higher mortality (30), anemia in liver transplant recipients may present additional challenges such as graft dysfunction and mortality (3). Due to the cross-sectional design of the study, clinical implications are difficult to assess. However, there was a borderline significant association between anemia and breathlessness. Further studies of the clinical presentation and clinical consequences of anemia in liver transplant recipients are warranted.

We found a prevalence of neutropenia of 2.1% in liver transplant recipients that were more than one-year post-transplantation. In contrast, previous studies have reported the prevalence of neutropenia ranging from 24% to 31% within the first-year post-transplantation (21, 36). The low prevalence of neutropenia among both liver transplant recipients and controls in our study precluded fully adjusted analyses of potential risk factors. However, neutropenia was still twenty times more prevalent among liver transplant recipients than controls. This increased prevalence of neutropenia may partly be explained by the use of immunosuppressive medications, due to bone marrow suppression and inhibition the production of neutrophil precursors (6). Existing evidence found an increased risk of infections, hematological malignancies, and mortality in neutropenic patients, with greater severity being associated with worse outcomes (37, 38).

Liver transplant recipients also had a higher prevalence of lymphocytopenia and more severe cases than controls, and living with a transplanted liver, higher hs-CRP levels and older age were independent risk factors for lymphocytopenia. Surprisingly, the specific types of immunosuppressive medications and prednisolone use were not associated with lymphocytopenia in our study, possibly due to confounding by indications. In our study, more than one in six liver transplant recipients had lymphocytopenia and among those with lymphocytopenia 10% had severe lymphocytopenia. Lymphocytopenia has been associated with increased susceptibility to infections (39–42) and worse cumulative survival in liver transplant recipients (43). While one study found lymphopenia to be associated with a 1.4-fold increased risk of infections and a 1.7-fold increased risk of mortality from infection in the general population (39). However, further studies are needed to assess the potential long-term impact of lymphocytopenia on morbidity and mortality in liver transplant recipients.

Liver transplant recipients had lower thrombocyte counts than controls and living with a transplanted liver was independently associated with higher odds of thrombocytopenia in our study. Older age and male sex were associated with higher odds of thrombocytopenia. Among liver transplant recipients, transplant-related risk factors such as specific types of immunosuppressive medications and cirrhosis at time of transplantation were not associated with thrombocytopenia. A previous study with serial measurement of thrombocytes found that the prevalence of thrombocytopenia declined from 54% one-year post-transplantation to 25% three-years post-transplantation (10), we found an even lower prevalence of thrombocytopenia (19.2%) among liver transplant recipients a median of 7.8 years after transplantation. However, we found no significant association between being 1–5 years post-transplantation compared to beyond 5 years post-transplantation and thrombocytopenia suggesting the effect of thrombocytopenia wanes before the fifth-year post-transplantation. Several factors may contribute to thrombocytopenia in liver transplant recipients, including reduced thrombocyte lifespan and reduced thrombocyte production. This could be due to a reduced release of the growth factor, thrombopoietin, which under normal circumstances is secreted from the liver. Finally, factors such as hypersplenism may also contribute to the development of thrombocytopenia. Although most cases of thrombocytopenia were either mild or moderate in our study, lower thrombocyte counts may still be associated with a higher risk of bleeding and infections (6, 44). Studies investigating serum levels of thrombopoietin, or the thrombocyte function may provide additional information about the pathogenesis.

Autoimmune liver disease was the most common cause of transplantation in the DACOLT cohort which is typical for Scandinavian populations. To address generalizability, we conducted two stratified analyses where we only included liver transplant recipients with cirrhosis at time of transplantation and excluded liver transplant recipients with autoimmune liver disease. Our results were consistent, indicating generalizability across different causes of liver transplantation.

Furthermore, we found no significant differences in cytopenia prevalence between recipients with autoimmune liver disease and those transplanted for other reasons, suggesting that the increased risk is not related to a specific indication for transplantation, but rather a general consequence of living with a transplanted liver.

Our study had limitations: First, recall bias cannot be excluded, as some information was collected from questionnaires, albeit this bias would be present in both liver transplant recipients and controls. Second, the absence of certain parameters in the blood samples resulted in an incomplete classification of anemia. Third, we were unable to assess if any cytopenia was symptomatic. Fourth, the cross-sectional study design limits our ability to assess whether any of the cytopenias were present prior to transplantation. Fifth, the lack of kidney function in our analyses limited our ability to explore its impact, which could have helped to better understand the underlying mechanisms. Furthermore, the cross-sectional design of the study limits our ability to establish causality and to determine clinical consequences of the finding. However, our study also had several strengths: First, the large population in the DACOLT and CGPS and the use of identical standard operating procedures in both studies allow for consistency and comparability in data collection and analysis. Second, we addressed all cytopenias according to the Common Terminology Criteria for Adverse Events (CTCAE) version 5.0 to ensure a standardized approach for future studies. Finally, through the Shared Medication Record (FMK) and extensive journal entries, we have complete and thorough dataset of transplant-related variables in our cohort to eliminate potential selection and recall bias.

In conclusion, liver transplant recipients had a higher prevalence of cytopenias compared to the general population and living with a transplanted liver was independently associated with anemia, thrombocytopenia, and lymphocytopenia. Among liver transplant recipients, no association was found between cytopenias and specific types of immunosuppressive medication or cirrhosis at the time of transplantation. While our study revealed that almost 39% of liver transplant recipients experienced hematological abnormalities, additional research is needed to evaluate the clinical significance of cytopenia and the effectiveness of preventive measures in this patient population.

## Data Availability

The datasets presented in this article are not readily available because this study has the potential to determine prevalence, incidence and risk factors for development of comorbidity in liver transplant recipients and may be used to develop guidelines on screening, monitoring and long-term treatment of liver transplant recipients and thereby improve survival. Use of data will be confined to the study group, but potential collaborators or request for data can be submitted to dacolt.rigshospitalet@regionh.dk. Requests to access the datasets should be directed to DACOLT, dacolt.rigshospitalet@regionh.dk.
