# Supplementary material for: Living with a transplanted liver is associated with cytopenias: a nationwide cohort study
Source: Front Gastroenterol (Lausanne). 2025 Aug 6;4:1543618. doi: 10.3389/fgstr.2025.1543618 (PMC12952440; doi:10.3389/fgstr.2025.1543618)
Supplement: Supplementary file 1 [file Table1.pdf]

Supplemental data

Supplementary Table 1: Logistics regression analyses of risk factors associated with anaemia and lymphocytopenia.

Model 1: Odds ratio (95% Confidence Interval) from univariate logistic regression analyses.

Model 2: Adjusted odds ratio (95% Confidence Interval) from multiple logistic regression adjusted for liver transplantation status, age, sex, ethnicity, hs-CRP, smoking, and alcohol.

	Anemia				Lymphocytopenia			
Characteristics	Model 1	*p*-value	Model 2	*p*-value	Model 1	*p*-value	Model 2	*p*-value
**Liver transplantation (yes / no)**	9.17 [6.52 – 12.89]	<0.001	7.84 [5.04 – 12.18]	<0.001	14.56 [9.24 – 22.95]	<0.001	16.69 [9.56 – 29.12]	<0.001
**Age**	1.03 [1.02 – 1.04]	<0.001	1.03 [1.02 – 1.05]	<0.001	1.03 [1.01 – 1.04]	0.001	1.03 [1.01 – 1.05]	0.0016
**Sex (male vs female)**	1.80 [1.30 – 2.50]	<0.001	1.79 [1.23 – 2.62]	0.003	0.99 [0.57 – 1.46]	0.95	0.86 [0.54 – 1.37]	0.52
**Ethnicity**								
**Danish**	*Reference*		*Reference*		*Reference*		*Reference*	
**Other Scandinavian**	0.74 [0.31 – 1.73]	0.48	0.69 [0.26 – 1.84]	0.46	-	-	-	
**Other European**	1.03 [0.54 – 1.97]	0.92	0.89 [0.43 – 1.81]	0.74	1.64 [0.85 – 3.16]	0.14	1.22 [0.57 – 2.61]	0.61
**Turkish**	5.56 [1.39 – 22.26]	0.02	1.99 [0.44 – 9.00]	0.37	5.13 [1.08 – 24.42]	0.04	1.46 [0.27 – 7.94]	0.66
**Pakistani / Indian / Sri Lankan**	1.75 [0.21 – 14.87]	0.61	0.50 [0.06 – 4.46]	0.54	4.37 [0.49 – 38.54]	0.18	1.36 [0.14 – 12.99]	0.79
**Arab / Iranian**	7.69 [1.37 – 43.29]	0.02	3.42 [0.50 – 23.40]	0.21	-	-	-	
**Other (Asian, African, Greenlandic etc.)**	5.12 [1.94 – 13.48]	<0.001	2.20 [0.63 – 7.67]	0.22	2.02 [0.46 – 8.78]	0.35	1.11 [0.22 – 5.50]	0.90
**Hs-CRP**	1.04 [1.01 – 1.07]	0.009	1.02 [1.00 – 1.04]	0.11	1.06 [1.02 – 1.09]	0.001	1.05 [1.02 – 1.08]	0.0027
**Smoking**								
**Never**	*Reference*		*Reference*		*Reference*		*Reference*	
**Current**	1.05 [0.64 – 1.72]	0.84	0.98 [0.56 – 1.74]	0.95	0.52 [0.23 – 1.15]	0.11	0.56 [0.24 – 1.32]	0.19
**Former**	0.85 [0.59 – 1.21]	0.36	0.90 [0.60 – 1.36]	0.61	1.05 [0.69 – 1.59]	0.81	1.22 [0.75 – 1.99]	0.43
**Alcohol**								
**Daily**	*Reference*		*Reference*		*Reference*		*Reference*	
**Weekly**	1.00 [0.54 – 1.82]	0.99	0.74 [0.38 – 1.41]	0.35	1.49 [0.61 – 3.63]	0.38	1.10 [0.40 – 2.98]	0.85
**Monthly**	1.70 [0.93 – 3.09]	0.08	1.02 [0.53 – 1.95]	0.96	3.75 [1.61 – 8.71]	0.002	2.15 [0.83 – 5.63]	0.12
**Never**	4.80 [2.76 – 8.37]	<0.001	1.03 [0.52 – 2.03]	0.93	6.45 [2.83 – 14.71]	<0.001	1.01 [0.37 – 2.74]	0.98

Supplementary Table 1: Logistics regression analyses of risk factors associated with thrombocytopenia (continued).

Model 1: Odds ratio (95% Confidence Interval) from univariate logistic regression analyses.
Model 2: Adjusted odds ratio (95% Confidence Interval) from multiple logistic regression adjusted for liver transplantation status, age, sex, ethnicity, hs-CRP, smoking, and alcohol.

	Thrombocytopenia			
Characteristics	Model 1	*p*-value	Model 2	*p*-value
**Liver transplantation (yes / no)**	10.30 [6.92 – 15.33]	<0.001	10.19 [6.07 - 17.13]	<0.001
**Age**	1.00 [0.99 – 1.01]	0.99	1.01 [0.99 – 1.02]	0.35
**Sex (male vs female)**	1.84 [1.26 – 2.68]	0.002	1.84 [1.19 – 2.84]	0.006
**Ethnicity**				
**Danish**	*Reference*		*Reference*	
**Other Scandinavian**	0.56 [0.17 – 1.80]	0.33	0.38 [0.09 – 1.63]	0.19
**Other European**	1.51 [0.81 – 2.83]	0.20	0.87 [0.40 – 1.92]	0.73
**Turkish**	1.66 [0.21 – 13.09]	0.63	0.53 [0.06 – 4.43]	0.56
**Pakistani / Indian / Sri**	10.32 [2.26 – 47.05]	0.003	3.14 [0.62 – 15.94]	0.17
**Lankan**	2.73 [0.32 – 23.39]	0.36	1.50 [0.16 – 14.24]	0.72
**Arab / Iranian**	2.56 [0.75 – 8.76]	0.14	1.78 [0.46 – 6.82]	0.40
**Other (Asian, African,**				
**Greenlandic etc.)**				
**Hs-CRP**	1.00 [0.98 – 1.03]	0.76	0.94 [0.87 – 1.01]	0.01
**Smoking**				
**Never**	*Reference*		*Reference*	
**Current**	0.72 [0.38 – 1.34]	0.30	0.62 [0.30 – 1.28]	0.20
**Former**	0.84 [0.55 – 1.25]	0.37	0.88 [0.55 – 1.41]	0.60
**Alcohol**				
**Daily**	*Reference*		*Reference*	
**Weekly**	0.75 [0.36 – 1.59]	0.46	0.54 [0.24 – 1.23]	0.14
**Monthly**	1.60 [0.79 – 3.24]	0.19	0.92 [0.42 – 1.99]	0.82
**Never**	4.21 [2.18 – 8.12]	<0.001	0.98 [0.44 – 2.16]	0.95

Supplementary Table 2: Linear analyses for hemoglobin, neutrophil granulocytes, lymphocytes and thrombocytes.

Model: Fully adjusted model for age, sex, ethnicity, hs-CRP, smoking, and alcohol.

	Hemoglobin		Neutrophile granulocytes	
Characteristics	Model	*p*-value	Model	*p*-value
**Intercept**	8.59 [8.43 – 8.75]	< 0.001	3.70 [3.37 – 4.03]	< 0.001
**Liver transplantation (yes / no)**	-0.28 [-0.37 – -0.20]	< 0.001	-0.33 [-0.51 – -0.15]	0.0003
**Age**	-0.00 [-0.01 – 0.00]	0.002	0.00 [-0.00 – 0.01]	0.25
**Sex (male)**	0.81 [0.75 – 0.87]	< 0.001	-0.08 [-0.20 – 0.04]	0.19
**Ethnicity**				
**Danish**	*Reference*		*Reference*	
**Other Scandinavian**	-0.08 [-0.22 – 0.05]	0.24	-0.04 [-0.32 – 0.25]	0.81
**Other European**	0.08 [-0.03 – 0.19]	0.15	-0.02 [-0.25 – 0.21]	0.87
**Turkish**	-0.26 [-0.66 – 0.13]	0.19	-0.04 [-0.85 – 0.76]	0.92
**Pakistani / Indian / Sri Lankan**	0.33 [-0.16 – 0.82]	0.19	0.17 [-0.92 – 1.25]	0.77
**Arab / Iranian**	-0.07 [-0.56 – 0.42]	0.78	-0.77 [-1.78 – 0.24]	0.14
**Other (Asian, African, Greenlandic etc.)**	-0.26 [-0.53 – 0.02]	0.07	0.23 [-0.33 – 0.78]	0.43
**Hs-CRP**	-0.00 [-0.01 – -0.00]	0.05	0.05 [0.04 – 0.06]	< 0.001
**Smoking**				
**Never**	*Reference*		*Reference*	
**Current**	0.09 [0.00 – 0.18]	0.05	0.66 [0.47 – 0.84]	< 0.001
**Former**	0.03 [-0.03 – 0.10]	0.32	0.02 [-0.11 – 0.15]	0.78
**Alcohol**				
**Daily**	*Reference*		*Reference*	
**Weekly**	-0.03 [-0.12 – 0.07]	0.58	-0.11 [-0.30 – 0.08]	0.25
**Monthly**	0.00 [-0.09 – 0.10]	0.93	0.07 [-0.13 – 0.26]	0.51
**Never**	-0.01 [-0.12 – 0.10]	0.86	0.13 [-0.09 – 0.35]	0.24

Supplementary Table 2: Linear analyses for hemoglobin, neutrophil granulocytes, lymphocytes and thrombocytes (Continued)

Model: Fully adjusted model for age, sex, ethnicity, hs-CRP, smoking, and alcohol.

	Lymphocytes		Thrombocytes	
Characteristics	Model	*p*-value	Model	*p*-value
**Intercept**	2.33 [2.16 – 2.51]	< 0.001	261.61 [247.45 – 275.77]	< 0.001
**Liver transplantation (yes / no)**	-0.45 [-0.54 – -0.35]	< 0.001	-38.59 [-46.14 – -31.04]	< 0.001
**Age**	-0.00 [-0.01 – 0.00]	0.0001	-0.10 [-0.29 – 0.10]	0.33
**Sex (male)**	-0.11 [-0.17 – -0.05]	0.0006	-24.40 [-29.48 – -19.32]	< 0.001
**Ethnicity**				
**Danish**	*Reference*		*Reference*	
**Other Scandinavian**	0.00 [-0.14 – 0.15]	0.96	3.61 [-8.45 – 15.66]	0.56
**Other European**	0.01 [-0.11 – 0.13]	0.93	-5.05 [-14.86 – 4.76]	0.31
**Turkish**	-0.20 [-0.62 – 0.23]	0.36	-17.24 [-51.75 – 17.28]	0.33
**Pakistani / Indian / Sri Lankan**	0.25 [-0.32 – 0.82]	0.39	-23.06 [-66.31 – 20.20]	0.30
**Arab / Iranian**	0.07 [-0.45 – 0.60]	0.78	4.67 [-38.47 – 47.81]	0.83
**Other (Asian, African, Greenlandic etc.)**	0.15 [-0.14 – 0.45]	0.31	18.11 [-5.76 – 41.99]	0.14
**Hs-CRP**	-0.00 [-0.01 – 0.00]	0.51	1.03 [0.61 – 1.45]	< 0.001
**Smoking**				
**Never**	*Reference*		*Reference*	
**Current**	0.36 [0.26 – 0.46]	< 0.001	17.40 [9.48 – 25.31]	< 0.001
**Former**	0.03 [-0.04 – 0.10]	0.35	2.65 [-2.94 – 8.24]	0.35
**Alcohol**				
**Daily**	*Reference*		*Reference*	
**Weekly**	-0.03 [-0.13 – 0.06]	0.51	-0.82 [-8.79 – 7.15]	0.84
**Monthly**	0.00 [-0.10 – 0.10]	0.98	-5.75 [-14.16 – 2.66]	0.18
**Never**	0.09 [-0.03 – 0.21]	0.13	-3.22 [-12.66 – 6.22]	0.50

Supplementary Table 3: Odds ratios for cytopenias comparing liver transplant recipients with cirrhosis at time of transplantation with controls.

Model 1: Base model adjusted for age and sex.
Model 2: Fully adjusted model for age, sex, ethnicity, hs-CRP, smoking, and alcohol.

	OR for cytopenias comparing liver transplant recipients with cirrhosis at time of liver transplantation to controls			
	**Model 1 OR (95% CI)**	***p*-value**	**Model 2 OR (95% CI)**	***p*-value**
**Anemia**	9.53 [6.54 – 13.87]	<0.001	7.65 [4.66 – 12.56]	<0.001
**Neutropenia**	42.84 [5.11 – 358.94]	<0.001		
**Lymphocytopenia**	15.51 [9.51 – 25.29]	<0.001	18.44 [9.92 – 34.28]	<0.001
**Thrombocytopenia**	10.88 [7.05 – 16.79]	<0.001	11.68 [6.53 – 20.90]	<0.001

Supplementary Table 4: Odds ratios for cytopenias comparing liver transplant recipients without autoimmune liver disease as reason for transplantation with controls.

Model 1: Base model adjusted for age and sex.
Model 2: Fully adjusted model for age, sex, ethnicity, hs-CRP, smoking, and alcohol.

	OR for cytopenias comparing liver transplant recipients without autoimmune liver diseases to controls			
	**Model 1 OR (95% CI)**	***p*-value**	**Model 2 OR (95% CI)**	***p*-value**
**Anemia**	8.34 [5.52;12.61]	<0.001	7.47 [4.24;13.18]	<0.001
**Neutropenia**	58.52 [7.14;479.60]	<0.001		
**Lymphocytopenia**	14.72 [8.83;24.56]	<0.001	21.10 [10.90;40.84]	<0.001
**Thrombocytopenia**	10.99 [6.99;17.28]	<0.001	13.20 [7.00;24.92]	<0.001

Supplementary Table 5: Associations between tacrolimus trough levels and cytopenias in liver transplant recipients.

Logistic regression models for neutropenia were not feasible due to very few events.

Model 1: Base model adjusted for age and sex.

Model 2: Fully adjusted model for age, sex, ethnicity, hs-CRP, smoking, and alcohol.

	OR for cytopenias comparing liver transplant recipients with cirrhosis at time of liver transplantation to controls			
	**Model 1 OR (95% CI)**	***p*-value**	**Model 2 OR (95% CI)**	***p*-value**
**Anemia**	0.43 [0.16–1.16]	0.094	0.36 [0.07–1.95]	0.24
**Neutropenia**	-	-	-	-
**Lymphocytopenia**	2.86 [0.70–11.81]	0.15	6.94 [0.71–67.62]	0.10
**Thrombocytopenia**	0.88 [0.32–2.44]	0.81	0.32 [0.04–2.27]	0.25
**Any cytopenias**	0.55 [0.22–1.38]	0.20	0.30 [0.06–1.47]	0.14
